# Identifying multi-layer gene regulatory modules from multi-dimensional genomic data

**DOI:** 10.1093/bioinformatics/bts476

**Published:** 2012-08-03

**Authors:** Wenyuan Li, Shihua Zhang, Chun-Chi Liu, Xianghong Jasmine Zhou

**Affiliations:** ^1^Program in Molecular and Computational Biology, Department of Biological Sciences, University of Southern California, Los Angeles, CA 90089, USA, ^2^National Center for Mathematics and Interdisciplinary Sciences, Academy of Mathematics and Systems Science, Chinese Academy of Sciences, Beijing 100190, China and ^3^Institute of Genomics and Bioinformatics, National Chung Hsing University, Taichung 402, Taiwan, Republic of China

## Abstract

**Motivation:** Eukaryotic gene expression (GE) is subjected to precisely coordinated multi-layer controls, across the levels of epigenetic, transcriptional and post-transcriptional regulations. Recently, the emerging multi-dimensional genomic dataset has provided unprecedented opportunities to study the cross-layer regulatory interplay. In these datasets, the same set of samples is profiled on several layers of genomic activities, e.g. copy number variation (CNV), DNA methylation (DM), GE and microRNA expression (ME). However, suitable analysis methods for such data are currently sparse.

**Results:** In this article, we introduced a sparse Multi-Block Partial Least Squares (sMBPLS) regression method to identify multi-dimensional regulatory modules from this new type of data. A multi-dimensional regulatory module contains sets of regulatory factors from different layers that are likely to jointly contribute to a local ‘gene expression factory’. We demonstrated the performance of our method on the simulated data as well as on The Cancer Genomic Atlas Ovarian Cancer datasets including the CNV, DM, ME and GE data measured on 230 samples. We showed that majority of identified modules have significant functional and transcriptional enrichment, higher than that observed in modules identified using only a single type of genomic data. Our network analysis of the modules revealed that the CNV, DM and microRNA can have coupled impact on expression of important oncogenes and tumor suppressor genes.

**Availability and implementation:** The source code implemented by MATLAB is freely available at: http://zhoulab.usc.edu/sMBPLS/.

**Contact:** xjzhou@usc.edu

**Supplementary information:** Supplementary material are available at *Bioinformatics* online.

## 1 INTRODUCTION

Eukaryotic gene expression (GE) is a complex process controlled at multiple levels, including epigenetic, transcriptional and post-transcriptional regulation. Dynamic and precise coordination of these regulatory processes is essential to maximize the efficiency and specificity in GE. Recent studies support the view that, rather than a simple ‘step-by-step’ production line, the GE machine is governed by multiple, complex and extensively coupled networks (Maniatis and Reed, 2002; Moore, 2005; Orphanides and Reinberg, 2002).

The development of high-throughput genomic technologies has enabled researchers to obtain a global view of gene regulation. Microarray and sequencing technologies can not only measure genome-wide GE levels but also profile DNA modifications (e.g. CNV [copy number variation]), epigenetic regulation (e.g. DNA methylation [DM] and histone modifications) and post-transcriptional regulation (e.g. microRNA expression [ME]). However, most genome-wide studies have been restricted to only one aspect of regulation such as studies based on GE profiles (Alter *et al.*, 2000; Omberg *et al.*, 2007; Tamayo *et al.*, 2007). Recently, a new type of large-scale multi-dimensional genomic dataset has been gaining in popularity. In these datasets, the same set of samples is profiled on several layers of genomic activity, e.g. CNV, DM, GE and ME. The Cancer Genomic Atlas (TCGA) (McLendon *et al.*, 2008) project and the NCI60 (Shoemaker, 2006) project provide this type of comprehensive genomic characterization for a cohort of cancer samples and cancer cell lines, respectively. Multi-dimensional datasets provide unprecedented opportunities to discover connections between the different layers of GE regulation.

The emerging large-scale multi-dimensional genomic data calls for novel computational methods. In fact, as the cost of sequencing falls, multi-dimensional characterization of samples will soon become standard practice. However, suitable analysis methods are currently sparse. In particular, since the different types of genomic data have different scales and units, we cannot simply aggregate them for analysis. Previous relevant effort has mostly focused on two-dimensional genomic datasets. For example, various eQTL methods can jointly analyze single-nucleotide polymorphism (SNP) and GE data to identify regulatory SNPs (Zhang *et al.*, 2010); multivariate regression can correlate GE and transcription factor (TF) binding data to associate TFs with their target genes (Gao *et al.*, 2004); and the Ping-Pong algorithm integrates GE and drug–response data (Kutalik *et al.*, 2008). Recently, several methods have been developed to analyze genomic datasets with more than two dimensions. For example, the multivariate model developed by Mankoo *et al.* (2011) and the sparse regression method proposed by Witten and Tibshirani (2009) , both can learn multi-dimensional genomic data in the supervised manner. Another relevant method, cMonkey, is a multi-species biclustering method that was applied to analyze GE matrices from different species (Waltman *et al.*, 2010). In addition, there have been a series of multiple kernel learning methods designed for integrating heterogeneous genomic data (Alpaydin, 2011; Hamid *et al.*, 2012; Yu *et al.*, 2010). These methods combine multiple kernels (each of which is transformed from a data type) into a single kernel, known as kernel fusion, which is then used for prediction, regression or feature selection.

**Fig. 1.**
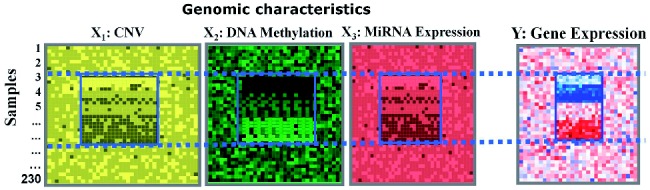
Illustration of a ‘multi-dimensional regulatory module’. A subsets of CNVs, DMs and MEs exhibit similar profiles as a subset of GEs across a subset of samples

In this article, we used a novel approach for supervised module discovery from *R*-dimensional genomic data (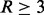), a topic that was not covered by the aforementioned methods. In particular, we aim to identify multi-dimensional gene regulatory modules, which from *R*-dimensional genomic data (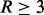). In this application, a regulatory module contains sets of regulatory factors from different layers that are likely to contribute jointly to a local ‘gene expression factory’. Without losing generality, assume that we are given a four-dimensional dataset consisting of GE, copy number variation (CNV), DNA methylation (DM) and microRNA expression (ME) profiles measured on the same *K* samples (Figure 1). Considering CNV, DM and ME as the input variables and GE as the response variable, we represent this dataset as four matrices: 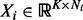, where *i* = 1, 2, 3 and 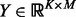. In each matrix the rows correspond to the same samples. The columns of the matrices correspond to measurements of different types. We aim to identify subsets of the three types of variables (CNV, DM and ME) that jointly explain the expression of a subset of genes, in all or a subset of the samples. The union of these four subsets of the different types of variables are termed a ‘Multi-Dimensional Regulatory Module (MDRM)’ (Figure 1). In our matrix representation, such a module consists of *k* rows, and 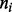 (*i* = 1, 2, 3) and *m* columns for the CNV, DM, ME and GE data. This approach captures the association between different types of variables (CNV–DM–ME) in terms of their joint impact on GE and facilitates the reconstruction of the regulatory network across different layers.

To identify multi-dimensional regulatory modules, we introduced a sparse Multi-Block Partial Least Squares (sMBPLS) regression method. Partial least squares (PLS) is a class of regression methods for finding the fundamental relations between an input matrix (*X*) and a response matrix (*Y*). Instead of finding hyperplane of maximum variance between the input and response variables, it finds a linear regression model by projecting both variables to a new space (Boulesteix and Strimmer, 2007; Fornell and Bookstein, 1982; Lê Cao *et al.*, 2008; Liu and Rayens, 2007; Tenenhaus *et al.*, 2005). MBPLS method is an expansion of the PLS for the regression analysis of input variables that are blocked into multiple subsections (Wangen and Kowalski, 1988; Wold *et al.*, 1987). MBPLS was initially developed for the chemometrics analysis and has been rarely applied to Bioinformatics (Hwang *et al.*, 2004; Li and Chan, 2009). The multi-dimensional genomic data provide a new opportunity for its application. In this article, we further expanded the MBPLS method by imposing sparse constraints to identify multi-dimensional modules. In particular, we imposed sparse constraints in both genomic variables and the sample dimensions. Different from the original MBPLS whose objective is the regression analysis of the whole data blocks, the sMBPLS aims to decompose the whole data blocks into a collection of smaller building blocks—MDRMs.

We demonstrated the performance of our method on both simulated data and the multi-dimensional TCGA datasets. The simulation study showed that the sMBPLS method can accurately identify embedded multi-dimensional modules and remarkably outperforms the non-sparse approach. We applied sMBPLS method to a suite of TCGA data including the CNV, DM, ME and GE data on 230 ovarian cancer samples. We showed that majority of identified modules have significant functional and transcriptional enrichment, higher than that observed in modules identified using only a single type of genomic data. Our network analysis of the modules revealed that the multi-dimensional genomic components are tightly connected and the CNV, DM and microRNA can have combinatorial impact on expression of important oncogenes and tumor suppressor genes. Finally, we compared our sMBPLS approach to the commonly used approach in which all input data blocks were aggregated into a single block for module discovery. We found that almost half of modules from the single-block approach are not multi-dimensional, demonstrating the importance of our ‘multi-block’ approach in capturing functional relationships of variables from multiple dimensions.

## 2 METHODS

### 2.1 Definition of the MDRM

Given three input blocks 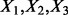 and a response block *Y*, a multi-dimensional module is defined by satisfying the criterion “the profiles extracted from 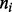 columns across k rows of 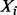 *(i = 1, 2, 3)* has strong association with (or has similar and coherent pattern with) those from *m* columns across the same *k* rows of *Y*” (Fig. 1). Such association between two submatrices from 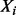 and *Y* can be measured by the covariance of their ‘summary vectors’. The coincidence of these associations appearing in the same *k* samples establishes the strong signals that multiple types of input variables explain the response variables. Such distinct covariance structure in subspaces of multiple blocks can be identified by the sparse version of the MBPLS regression framework.

### 2.2 Objective function

**Fig. 2.**
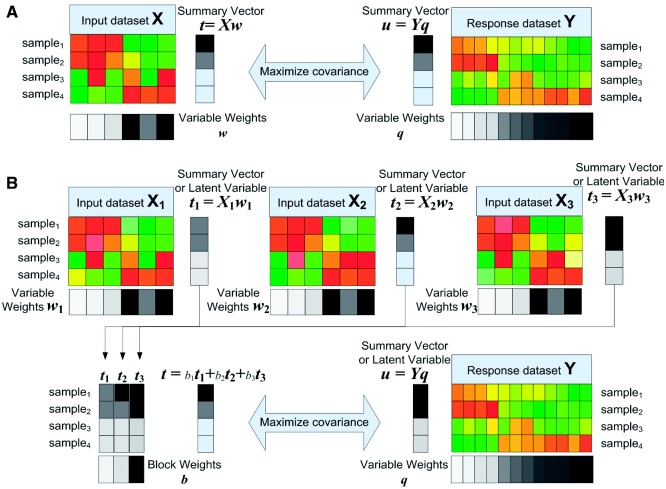
Illustration of (**A**) the covariance function for measuring the association of two matrices and (**B**) the problem formulation of multi-dimensional module discovery. To search a multi-dimensional module, columns of each block are represented by a ‘summary’ vector, e.g. 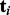 summarizing 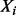 and **u** summarizing *Y*. Then the association between each input dimension 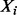 and the response dimension *Y* is measured by the covariance of their each summary vectors, i.e. 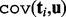. The maximum covariance between summary vectors of 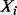 and *Y* reveals a distinct association representing the coherent profiles of 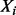 and *Y*. The maximization can be achieved by how we construct the summary vectors by weighting variables and samples. This discovery process is equivalent to the sparse version of the MBPLS problem

We first introduce the covariance function of measuring the association between two matrices. Let *X* and *Y* be input and response matrices on the same *K* samples, respectively. To summarize columns of a matrix *X*, we introduce a ‘summary’ vector **t** which is a linear combination of all columns of *X*, i.e. **t** = *X* **w** (**w** being the weights of input variables/columns). Similarly, **u** is a summary vector of *Y* columns, i.e. **u** = *Y* **q** (**q** being the weights of response variable/columns). Thus, the larger the covariance of two summary vectors *t* and *u* are, the more similar two matrices look like and the higher the association of two matrices is (Figure 2A). This association measure can be extended to multiple blocks of input variables.

Consider three input blocks 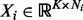 (where *i* = 1, 2, 3), each of which contains a set of 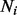 centered (zero-mean) input variables on the same *K* samples, and let 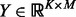 be the response data with *M* centered variables on the same *K* samples (Figure 2B). We used the weighted sum 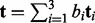 of ‘summary vectors’ 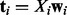 (*i* = 1, 2, 3) of the three sets of input variables. The block weights 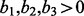 indicate the contribution of each data block to the covariance structure of the input and response data. Therefore, the covariance between **t** and **u** = *Y* **q** measures the association between three input data blocks and a response data block. The maximization of covariance between **t** and **u** can reveal the associations between from 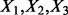 and *Y*, which lead to the discovery of a multi-dimensional module (Figure 2B). The problem is formally expressed as follows:
(1)
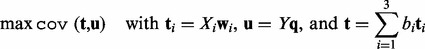

subject to 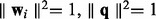, and 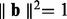. This is also the objective of the MBPLS regression problem (Wangen and Kowalski, 1988) which seeks to best explain the covariance structure between multiple groups of input variables and response variables. 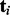 and **u** are also called the ‘latent variables’ of the *i*^th^ block 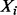 and block *Y*, respectively, and 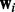 and **q** are their associated ‘loading vectors’.

Although the solution to this objective can identify a multi-dimensional module by selecting those variables and samples with large absolute values from 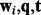, such module may not be the most distinct. As shown in our simulation study, the MBPLS regression approach often fails to identify distinct association signals of coherent structures. To address this problem, we added the sparsity penalties to the above objective.

### 2.3 Sparsity penalization

Sparsity penalization has recently attracted intense interest in regression analysis (Friedman, 2008), variable selection (Chun and Keles, 2010; Lê Cao *et al.*, 2008), matrix factorization (Kim and Park, 2007; Shen and Huang, 2008) and module discovery. The concept of sparsity (also called sparse coding in the literature) refers to a representational scheme (e.g. loading vector) where only a few elements are effectively used to represent data. Such sparsity is attractive from a data analysis viewpoint and makes representational scheme easy to interpret, as it selects the important elements and discards the rest. In effect, this implies most elements taking values close to zero while only few take significantly non-zero values. In our sparse version of the MBPLS problem, we searched the sparse representations of loading vectors whose non-zero elements can form a multi-dimensional module. It is achieved by adding sparsity penalties or regularizations to the optimization problem. Specifically, we adopted the widely used ‘lasso penalization’ (Tibshirani, 1996), which has been successfully applied in many fields. Let **x** be the vector to be computed in the optimization problem. The lasso regularization of **x**, denoted 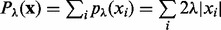 can be added to enforce sparsity on the solution of **x**. Our maximization problem becomes
(2)
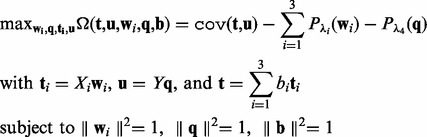

where the objective function 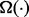 contains sparsity penalizations of the loading vectors 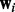 (*i* = 1, 2, 3) and **q**.

### 2.4 Sparse multi-block PLS algorithm

To solve the problem in equation (2), we propose a sparse multi-block PLS (sMBPLS) regression algorithm. In this algorithm, sparse() is the soft thresholding function 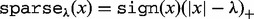 that is used to optimize the objective function with lasso penalties 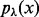. We prove that the sMBPLS algorithm can provide the maximizer of the sparse multi-block PLS problem (see Supplementary material).

**sMBPLS Algorithm**

1. Initialize: Apply the MBPLS algorithm to 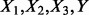 and obtain the latent variable 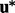. Set 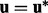.
2. Update: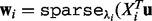), norm 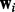 (*i* = 1, 2, 3)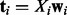 (or 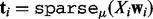) (*i* = 1, 2, 3)
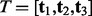

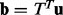
, norm **b**
  - **t** = *T*** b**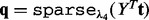, norm **q**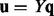 (or 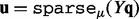)
3. Repeat Step 2 until convergence of **t**.

In this algorithm, **u** is regressed on each block 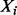 to give the loading vector 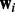 of the block, which are then multiplied through the block to provide the latent variable 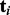. All three latent variables 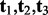 are combined into the super block *T* and a classic PLS iterative cycle between *T* and *Y* is performed to give the block weights **b** and the combined latent variable **t**. We repeat this until convergence on **t**. The computational complexity of one sMBPLS iteration is 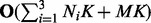. We are also interested in selection over the sample dimension. Specifically, we want to identify a multi-dimensional module whose input variables have the maximum covariance with response variables across a subset of samples. To achieve this goal, we impose the 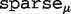 function on Step 2(b) and Step 2(g) of the sMBPLS algorithm to select samples.

The iterative procedure of the sMBPLS algorithm can be used to obtain the first set of sparse loading vectors 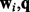 and latent variables **t**. The non-zero elements of converged loading vectors and latent variable (**t**) identify a multi-dimensional module that contains subsets of input and response variables and a subset of samples. After we identify a module, we deflate the matrix by subtracting the signal of current set of loadings and latent variables from the data matrices. Subsequent modules, or subsequent sets of sparse loadings and latent variables, can be obtained sequentially by maximizing covariance on the deflated matrices. We used the following deflation formula to remove the module’s signal from each block:

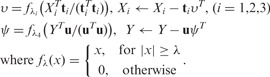



### 2.5 Tuning parameter selection

The sMBPLS algorithm is developed for fixed 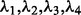 (with the option of including 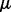). We tune these parameters using the cross-validation procedure that was also used in recently proposed methods such as sparse PCA (Shen and Huang, 2008) and sparse PLS (Lê Cao *et al.*, 2008). Tuning these parameters is equivalent to choosing the ‘degree of sparsity’, i.e. the number of non-zero components in each loading vector and latent variable. Note that setting the degree of sparsity to *j* (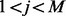) (taking the loading vector **q** as an example) is equivalent to setting 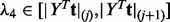, where 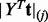 is the *j*^th^-order statistic of 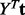. Our computational framework allows different vectors to have different degrees of sparsity. To simplify notations, we use 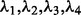 and 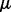 to denote the degrees of sparsity of loading vectors and latent variables in the rest of the article. The cross-validation procedure is presented below.

1. Randomly place the samples into *L* roughly equal groups. Each group has a corresponding matrix from each block of data. That is, the matrix of the *i*^th^ genomic block data with only samples in the *l*^th^ group is denoted 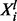; let 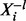 be the matrix composed of data from all other samples. The same notation applies to the response block data *Y*, which is divided into 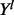 and 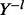 for each group.
2. For each combination of degrees of sparsity, 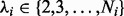 (i = 1,2,3), 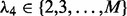 and 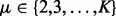  - For 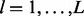, apply the sMBPLS algorithm on 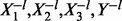, to derive loading vectors of independent variables: 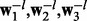 and the loading vector of response variables 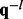. Next, project 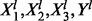 onto 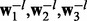 and 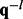 to obtain the projection coefficients as 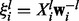 and 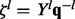, respectively.
  - Calculate the *L*-fold CV score defined as
(3)
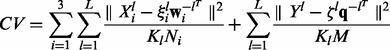

where 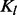 is the number of samples in the *l*th group.
3. Select the combination of degrees of sparsity 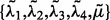 whose CV score is the smallest.

*L* and the number of combinations of degrees of sparsity will impact the computational efficiency. In practice, *L* is usually chosen to be 5 or 10 for large datasets. Naturally, a different random grouping in Step 1 may result in different degrees of sparsity. Usually, the larger the value of *L*, the more stable the degrees of sparsity selected by CV. In our study, we use *L* = 5, which is large enough for a stable selection of parameters. The number of combinations of degrees of sparsity for the thresholds 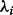 (*i* = 1, 2, 3, 4) and 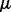 is large for large-scale data. Therefore, in practice, we used a subset of combinations in the cross-validation procedure.

## 3 RESULTS

To assess the performance of sMBPLS, we first applied the sMBPLS to a variety of simulated data, generated with various complexities. We then applied sMBPLS to the multi-dimensional TCGA ovarian cancer data to gain insights into the multi-layer coordination of GE regulations. To reveal the advantages of sMBPLS, we also compared our methods to several competing methods including MBPLS, sparse PLS and biclustering methods.

### 3.1 Simulation study

The simulation data were generated by extending scenarios proposed in the recent literature on sparse PLS methods (Chun and Keles, 2010; Lê Cao *et al.*, 2008) to multi-dimensional data (see Supplementary material for details). The scenario follow the general model of 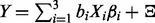, where 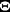 is the Gaussian noise and the settings of 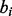 and 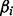 refer to the Supplementary material. 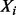 is generated with various complexity: different sizes, hidden component structures, correlation structures and even the presence of multicolinearity (simulated from a multivariate normal with a first-order autoregressive process’s covariance matrix with auto-correlation 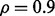).

In order to discover the multi-dimensional modules by using MBPLS, an intuitive two-step procedure can be performed: first applying MBPLS to the data, then selecting the top-ranking input and response variables to form a module by ordering the absolute values of the loadings and weight vectors. A more intuitive comparison way is to sort the loadings and latent variable from either MBPLS or sMBPLS, then to visualize 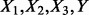 whose rows and columns are reordered by the sortings of their loadings and weight vectors. The multi-dimensional module would appear in the left-top and right-bottom corners (whose corresponding variables and samples have large absolute values of weights) in the reordered blocks. For example, the reordered blocks by MBPLS as shown in Figure S1(B)-panel2 (in Supplementary material) are observed to have no clear modular submatrices in corners, while a multi-dimensional module can be observed in reordered blocks by sMBPLS in Figure S1(B)-panel3 and zoomed out in Figure S1(C).

We further systematically compared two methods by simulating data 50 times. We found that MBPLS always failed for all 50 simulation data in that it assigns totally different variables and samples to the discovered module. In contrast, all modules identified by sMBPLS have significant overlaps with predefined modules. (Details of overlap significance test for modules can be found in Section S3 of Supplementary material.) An example is detailed in Figure S1. Since the MBPLS method maximizes the covariance between all input and response variables across all samples as shown in Figure S1(B), it overlooks embedded modules when the module’s signal is overwhelmed by background noise. On the contrary, the sparsity penalty forces sMBPLS to focus on ‘local’ (i.e. across a small subsets of variables and samples) peaks in the covariance, which correspond to (multi-dimensional) modules of relatively small size. Our results show that the sMBPLS method is more accurate at identifying modules in noisy data, and thus more suitable for biological applications.

We also compared the sMBPLS with the sparse version of PLS which is applied to the combined single input block (i.e. 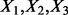 merged to a single block *X*). The same 50 times of simulation data were used. The result showed that all modules identified by sparse PLS have at least one dimension missing, 56% modules have two dimensions missing and none of these modules module has significant overlap with predefined module. The lack of such power for the single-block approach may attribute to the unbalanced covariance structures across multiple blocks, i.e. the covariance signals of some blocks may be overwhelmed by those of other blocks. The result is even worse when we compared with a popular biclustering algorithm ‘SAMBA’ (Tanay *et al.*, 2002) which is applied to the single block by merging 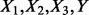. All modules identified by SAMBA have at least one dimension missing, and 84%/34% modules have at least two/three dimensions missing. None of these modules has overlaps with predefined modules.

### 3.2 Identifying MDRM in TCGA ovarian cancer data

We applied the sMBPLS method to the TCGA ovarian cancer genomic data. We included four types of genomic data profiled on the same 230 samples: CNV, DM, ME and GE. The data were downloaded from http://cancergenome.nih.gov/. Detailed preprocessing procedures can be found on the TCGA website (we used the Level 3 data). We filtered out genomic variables with little variation across the whole sample (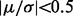, where 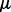 and 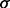 are the mean and standard deviation of each variable), resulting in a final dataset with the expressions of 799 microRNAs and 15 846 genes, the CNV profiles of 31 324 loci and the DMs of 14 735 marks (see Section S5 of Supplementary material). We repeated the iterative cycle of sMBPLS to identify the modules one by one, until no further significant gain is achieved on the covariances between input and response variables of the identified modules. In the following, we perform further analysis on the top 100 modules. The details of the 100 modules can be found on the supplementary website.

**Fig. 3.**
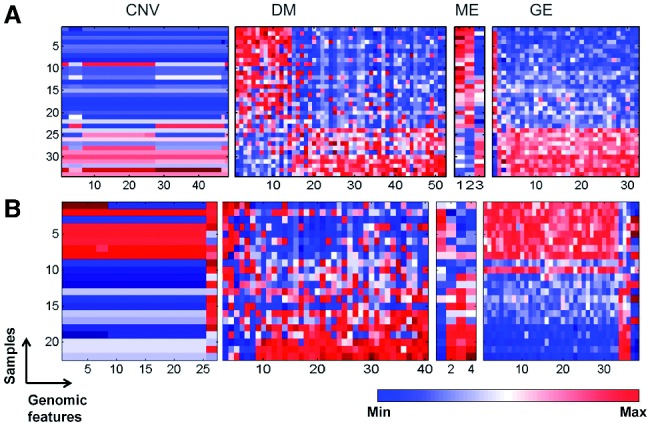
Heat map for feature profiles of CNV, DM, microRNA and GE in modules across the same small set of samples for (**A**) Module 23 and (**B**) Module 83. Each row represents a sample and each column represents a genomic feature

On average, each module contains 30 samples, 45 CNV loci, 42 methylation marks, 5 microRNAs and 44 genes (see the figure of the distribution of module sizes in Supplementary material). We used the overlap significance test on the identified 100 modules to investigate how distinct these modules are. Our results showed that only one pair of modules has significant overlap at the level of *P*-value after Bonferroni correction 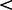
0.05 (see Section S8 of Supplementary material). Figure 3 shows the heat maps of two example modules, demonstrating the high degree of (anti-)correlation between the four dimensions. As expected, genomic profiles of most CNV marks are positively correlated with the expression levels of genes. But we can also see that genomic profiles of the methylation marks are sometimes partially anti-correlated with the expression levels of genes. We should note that our problem formulation shown in Figure 2(B) is the covariance maximization between **u** (from *Y*) and the weighted sum of 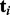 (from 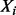), so it can well capture the holistic correlations between response block and all input blocks. This formulation can work well but cannot guarantee to identify those modules in which each of input dimensions is highly correlated with the response dimension. Therefore in some cases such as shown Figure 3(B), there does exist some input dimension that may not have expected correlation with the response dimension. In addition to identifying multiple types of variables that jointly explain the expression of a set of genes, sMBPLS also provides the relative weights (i.e. the block weights 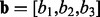) of each dimension in contributing to the observed covariance. The weight 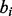 (*i* = 1, 2, 3) is proportional to the correlation of 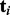 and **u** in the sMBPLS model. Among the 100 modules, we observed a significant correlation between the latent variables 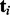 of the CNV, DM and ME dimensions and that **u** of the GE dimension in 63, 100 and 91 modules, respectively (*P*-value 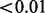, computed from a Student’s *t* distribution for a transformation of the Pearson’s correlation). In reality, it is not necessary for all dimensions to be equally important. The block weight information can help to identify important regulatory factors from the selected variables. To evaluate how robust these identified modules are, we randomly remove 10% samples from the dataset and then performed the same procedure to identify 100 modules. By using the overlap significance test, we checked the overlaps between them and the 100 modules identified from full set of samples. The 74 modules identified from 90% samples show significant overlaps over at least three dimensions with 79 modules identified from full set of samples. This result indicates good robustness of our method.

Because none of conventional methods can solve our problem, we could only resort to comparing with those well-established methods that can approximately arrive at our target. We used two classes of conventional methods: (1) biclustering algorithms, which identify correlated subsets of features across subsets of samples from a single block (e.g. combined *X* and *Y*) and (2) sparse PLS methods, which make regression analysis over two blocks *X* and *Y*. The former is unsupervised learning for exploratory data analysis while the latter is supervised learning. We used SAMBA, a popular adopted biclustering algorithm (Tanay *et al.*, 2002), to perform on the block *X* combining all four dimensions (CNV, DM, ME and GE). Out of the top 100 modules identified by SAMBA, 59% of the modules missed at least one type of variables and 22% of the modules missed at least two types of variables. We further compared our sMBPLS approach to the sPLS approach in which we combined three types of input data, CNV, DM and ME into a single combined block *X*. Out of the top 100 modules identified by sPLS, 47% of the modules missed at least one type of input variables and 17% of the modules were only one-dimensional. This result showed the unique advantage of multi-block modeling in capturing modules that elucidate relationships of variables from multiple dimensions.

### 3.3 MDRMs reveal synergistic functions across multiple dimensions

**Fig. 4.**
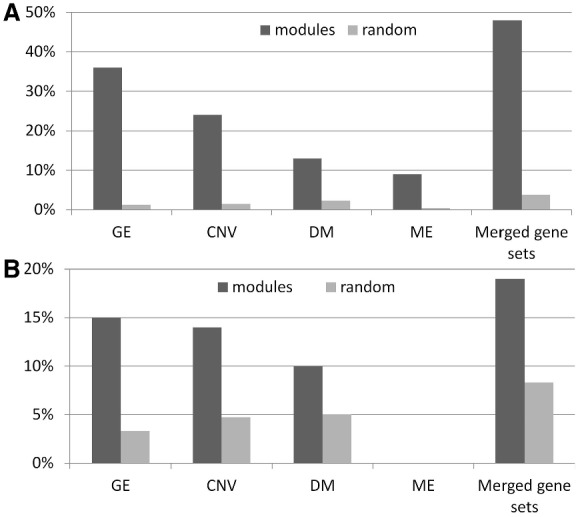
Comparison of **(A)** functional homogeneity and **(B)** transcriptional homogeneity between gene sets from identified modules (blue bars) and randomized gene sets (red bars). The gene set of an identified module is either from each individual dimension (GE, CNV, DM or ME) of the module or from genes combining all dimensions of the module. Shown are percentages of gene sets that are functionally or transcriptionally enriched with *q*-value 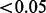

To evaluate the biological relevance of those identified multi-dimensional modules, we first test the functional homogeneity for each dimension of them. A set of genes is defined as functionally homogenous if it is enriched in at least one GO category (Ashburner *et al.*, 2000) with a *q*-value 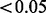 (the *q*-value is the *P*-value after a false discovery rate multiple testing correction). This was often the case. The GE dimension is functionally homogenous with respect to genes in 36% modules; the CNV dimension with respect to CNV-harbored genes in 24% modules; the DM dimension with respect to Methylation mark adjacent genes in 13% modules and the ME dimension with respect to microRNA in 9% modules (microRNA function was predicted based on the functions of their target genes), which are significantly higher than the 1.24%, 1.48%, 2.32% and 0.35% modules after randomization (Figure 4A), respectively.

Moreover, in 17 of those miRNA modules, miRNAs were enriched with members from the same miRNA clusters (*q*-value 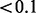 after multiple testing correction), where a miRNA cluster is defined as a set of miRNAs located within 50 kb in the genome (Baskerville and Bartel, 2005). miRNAs in a cluster are expected to play related functional roles (Yuan *et al.*, 2009). For example, the Module 96 includes five miRNAs (miR-let-7e, miR-125a, miR-150, miR-200c and miR-141). Two of these, let-7e and 125a, are members of a miRNA cluster in chromosome 19, while miR-200c and miR-141 belong to another miRNA cluster in chromosome 12. It can also be shown that many modules contain genes targeted by miRNAs in the same modules (see Section S6 in Supplementary material). Members of the let-7-family have been extensively reported to suppress ovarian cancers (Koturbash *et al.*, 2010). Also, miR-125a, miR-200c and miR-141 were reported to be dys-regulated in ovarian cancer. To take another example, Module 45 covers three miRNAs (miR-27a, miR-23a and miR-205). Interestingly, miR-27a and miR-23a are clustered in the genome and both have been reported to be up-regulated in ovarian cancer (Koturbash *et al.*, 2010). In addition, miR-205 has been extensively studied in relation to cancers of the bladder, lung, pancreatic, breast, esophagus and prostate.

While the individual dimensions of those modules already exhibit significant level of functional homogeneity, combining all dimensions reveals an even stronger functional synergy. When we consider all genes in the GE dimension, CNV-harbored genes, methylation adjacent genes and microRNAs, 48 out of the 100 modules were found to be functionally homogenous (Figure 4), compared to 3.8% modules in randomized data. This result highlights the power of multi-dimensional modules in grouping functionally relevant factors from different regulatory layers. In addition, we further compared the *q*-values of GO terms enriched for modules identified by either our method or the sparse PLS method by following the comparison procedure described in (Costa *et al.*, 2008; Ernst *et al.*, 2005). The result indicates that our method can identify more functionally homogeneous modules with more diverse GO terms (see Section S7 of Supplementary material). Many of the identified modules are enriched of the biological processes such as cell cycle, cell activation, immune system process and so on, implying their possible involvement in cancer. Also, they contain many important genes (or microRNAs) known to be related to ovarian cancer. For example, Module 37 includes four *HOX* family genes (*HOXB2*, *HOXB4*, *HOXB6* and *HOXB7*) that all have been extensively reported to be related with ovarian cancer (Cheng *et al.*, 2005; Ota *et al.*, 2009; Widschwendter *et al.*, 2009; Wu *et al.*, 2007). In addition, the module contains a DM mark adjacent to *HOXA9* that was reported to be significantly hypermethylated in ovarian cancer patients (Widschwendter *et al.*, 2009; Wu *et al.*, 2007). In addition, the member genes *FGF19*, *GAS2*, *BMP7*, *TNFSF11*, and *FGFR3* are all known to play important roles in tumor genesis and progression. In the Module 76, miR-214, known to be involved in cell cycle, has been reported to be dys-regulated in ovarian cancer (Iorio *et al.*, 2007; Nam *et al.*, 2008; Yang *et al.*, 2008), and *CDH13* is a potential epigenetic biomarker for ovarian cancer (Wu *et al.*, 2007).

We then test the transcriptional homogeneity for each dimension of the identified multi-dimensional modules. We used the 191 ChIP-seq profiles generated by the Encyclopedia of DNA Elements (ENCODE) consortium (Thomas *et al.*, 2007). This dataset includes the genome-wide binding of 40 TFs, 9 histone modification marks and 3 other markers (DNase, FAIRE and DM) on 25 different cell lines (see Supplementary material). These data provide a set of potential targets of regulatory factors. A set of genes is defined as ‘transcriptional homogenous’ if it is enriched in the targets for any regulatory factor with a *q*-value 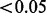. We achieved similar results as those of functional homogeneity analysis (Figure 4B). These modules are enriched of the TFs such as *SRF*, *STAT1* and *H3K27me3*. *SRF* regulates the activity of many immediate-early genes and thereby participates in cell cycle regulation, apoptosis, cell growth and cell differentiation. *STAT1* enhances inflammation and innate and adaptive immunity, triggering in most instances anti-proliferative and pro-apoptotic responses in tumor cells (Pensa *et al.*, 2009). Particularly, *STAT1* negatively regulates the cell cycle by inducing *p21 WAF1/CIP1* in ovarian cancer (Burke *et al.*, 1999). *H3K27me3* has been evaluated as a prognostic indicator for clinical outcome in patients with breast and ovarian cancers (Wei *et al.*, 2008).

### 3.4 MDRMs facilitate the regulatory analysis

Our method has provided sets of genomic features from different regulatory layers that are likely to be synergistic in their impact on GE. To further elaborate the relationships between those implicated features, we used the Ingenuity Pathway Analysis (IPA) system (Redwood City, CA, USA) to build molecular interaction networks. From each multi-dimensional module, we formed a set consisting of genes involved in the GE dimension, CNV-harbored genes, methylation-adjacent genes and microRNAs. Using this set as the input, IPA constructs networks based on literature-derived relationships between genes (or microRNAs) and computes a ranking score 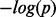 for each network. The *P*-value indicates the likelihood that the genes in the input network would be found together due to random chance. All of the multi-dimensional modules lead to statistically significant interaction networks (*P*-value 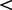 1.0E-20) by this analysis, which indicates the significant associations among them.

**Fig. 5.**
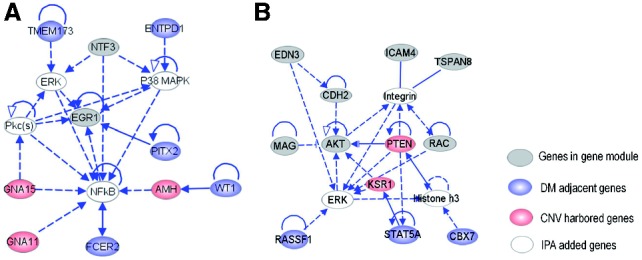
The molecular interaction networks (constructed by IPA) that center around the genes (**A**) *EGR1* and (**B**) *AKT*. The networks consist of affected genes (gray nodes), CNV-sharbored genes (red nodes) and DM-adjacent genes (blue nodes). The solid lines represent direct interactions and the dashed lines represent indirect interactions

Below, we provide in-depth descriptions of the heterogeneous regulatory networks that affect a key tumor suppressor gene (*EGR1*) and an oncogene (*AKT*) in ovarian cancer. *EGR1* is a cancer-suppressing gene known to be down-regulated in ovarian cancer (Lamber *et al.*, 2010). The network derived from the Module 4 reveals that complex connections of heterogeneous factors control the expression of *EGR1* (Figure 5A). A direct regulation on *ERG1* comes from *PITX2*, a gene adjacent to a DM mark in our module and known to be essential for the expression of *EGR1* in rat (Suh *et al.*, 2002). Multiple indirect influences on *EGR1* are transmitted by the TF *NFkB*, by *GNA15* and *GNA11* (genes hosted by CNVs in the module) and by *FCER2* (genes adjacent to the methylation marks in the module). In particular, *WT1* (regulated by DM) is known to positively regulate *AMH* (Nachtigal *et al.*, 1998) (which is also regulated by a CNV), which in turn positively regulates *EGR1* via *NFkB*. In addition, *EGR1* is regulated by both *TMEM173* and *ENTD1* (adjacent to methylations) via *ERK* and *P38 MARK*, respectively. The disruption of multiple neighbor nodes to *EGR1* by different regulatory mechanisms highlights the complex nature of the controls on this key suppressor gene for ovarian cancer. As another example, from Module 61 we derived a multi-layer coordinated network (Figure 5B) regulating *AKT*, a key oncogene for ovarian cancer (Altomare *et al.*, 2004; Yuan *et al.*, 2000) and an important component of the *PI3-kinase/AKT* pathway. The expression of *AKT* is positively regulated by the loss of a CNV-containing *PTEN*, which would otherwise down-regulate the expression of *AKT* (Pore *et al.*, 2003). Moreover, *AKT* is activated by *CDH2* (Rieger-Christ *et al.*, 2004), the expression of which, in turn, is increased by *EDN3* (Bagnato *et al.*, 2004). *AKT* is further up-regulated due to the methylation of *RASSF1* (a tumor suppressor gene), which is consistent with the previously observed over-expression of *AKT* in *RASSF1A*-depleted cells (Dallol *et al.*, 2005). Additional regulations are exerted by the loss of *KSR1* (CNV), the methylated *STAT5A* and the expression of *MAG*, among others. These factors, intertwined together, paint complex mechanisms leading to the activation of the important oncogene *AKT* in ovarian cancer.

In addition to the two examples detailed above, we observed multi-layer coupled regulatory networks around multiple oncogenes or tumor suppressor genes (e.g. *PIK3CA* and *CCNE1*). The results of this analysis clearly illustrate and illuminate the complex genetic origins of ovarian cancer. In fact, CNV, methylation and microRNA regulation are only few of the many expression regulatory mechanisms, and much more sophisticated coordination likely exists in the origins of this illness. The algorithm proposed in this study takes the first steps along the path to effectively integrate multi-dimensional data to explore the complex regulatory mechanisms.

## 4 CONCLUSIONS

In this study, we developed a sMBPLS regression method to identify multi-dimensional regulatory modules in diverse types of genomic data measured on the same set of samples. Classical eQTL analysis can only be applied to relate one type of genomic marker (e.g. SNP) to GE. In contrast, sMBPLS can identify combinations of multiple types of genomic markers that jointly impact the expression of a set of genes. We have applied the sMBPLS method to a suite of genomic profiles from 230 ovarian cancer samples, including CNV, DM, microRNA and GE data. The algorithm identified 100 modules, many of which display a high degree of functional homogeneity in at least one genomic dimension. If all dimensions of data are considered together, the modules exhibit an even greater degree of functional synergy. A detailed network view of individual modules reveals that many genomic features would remain isolated if we only considered one type of data. By combining diverse types of data, sMBPLS links the different regulatory layers and thus discovers more coherent and connected regulatory networks. Furthermore, our method derives weights for the dimensions of CNV, methylation and microRNA in each module, which indicate their relative contributions to the expression of individual sets of genes. We have demonstrated that multiple heterogeneous factors in a module can have combinatorial effects on GE. We should note that (1) this does not necessarily reflect the direct causal mechanisms for GE, but the revealed modules can be a good start point to further study the underlying mechanisms; (2) sMBPLS outperforms most existing algorithms in analyzing more than two data blocks, although it may not possibly improve the results when applied to only two blocks *X* and *Y*.

In summary, we expect that there will soon be a rapid increase of multi-dimensional data, and developing methods for such data will become an active research area. We have proposed a promising tool to extract coherent substructures from large-scale, complex datasets, greatly facilitating downstream biological analysis. Interpreting such complex modules is still a major challenge, given our limited knowledge of multi-layer coordination in biological systems. However, the rapid accumulation of multi-dimensional data and the knowledge derived from them will definitely accelerate a positive cycle of the knowledge discovery.
